# Phosphorylated protein chip combined with artificial intelligence tools for precise drug screening

**DOI:** 10.7555/JBR.37.20230082

**Published:** 2024-05-27

**Authors:** Katsuhisa Horimoto, Yuki Suyama, Tadamasa Sasaki, Kazuhiko Fukui, Lili Feng, Meiling Sun, Yamin Tang, Yixuan Zhang, Dongyin Chen, Feng Han

**Affiliations:** 1 SOCIUM Inc., Tokyo 1350064, Japan; 2 International Medical Center, Saitama Medical University, Saitama 350-1298, Japan; 3 Artificial Intelligence Research Center, National Institute of Advanced Industrial Science and Technology, Tokyo 1350064, Japan; 4 Department of Informatics and Data Science, Sanyo-Onoda City University, Yamaguchi 7560884, Japan; 5 International Joint Laboratory for Drug Target of Critical Illnesses, School of Pharmacy, Nanjing Medical University, Nanjing, Jiangsu 211166, China; 6 Department of Physiology, School of Basic Medical Sciences, Nanjing Medical University, Nanjing, Jiangsu 211166, China; 7 Gusu School, Nanjing Medical University, Suzhou, Jiangsu 215001, China; 8 National Vaccine Innovation Platform, Nanjing Medical University, Nanjing, Jiangsu 211166, China

**Keywords:** Phospho-Totum, protein array, signal transduction pathways, artificial intelligence tools, drug screening

## Abstract

We have developed a protein array system, named "Phospho-Totum", which reproduces the phosphorylation state of a sample on the array. The protein array contains 1471 proteins from 273 known signaling pathways. According to the activation degrees of tyrosine kinases in the sample, the corresponding groups of substrate proteins on the array are phosphorylated under the same conditions. In addition to measuring the phosphorylation levels of the 1471 substrates, we have developed and performed the artificial intelligence-assisted tools to further characterize the phosphorylation state and estimate pathway activation, tyrosine kinase activation, and a list of kinase inhibitors that produce phosphorylation states similar to that of the sample. The Phospho-Totum system, which seamlessly links and interrogates the measurements and analyses, has the potential to not only elucidate pathophysiological mechanisms in diseases by reproducing the phosphorylation state of samples, but also be useful for drug discovery, particularly for screening targeted kinases for potential drug kinase inhibitors.

## Introduction

The first step in cellular response to external stimuli is the process by which proteins (receptors) presented in the cell membrane are stimulated, and relevant information is then transferred to the nucleus. The biochemical background of intracellular signal transduction is the phosphorylation of proteins (substrates) by the kinases. It is well-known that molecules constituting intracellular signaling pathways responsible for cellular responses are candidate molecular targets for the treatment of cancers^[1–2]^, and a group of proteins in the correlation between kinases and their substrates have been investigated as the candidate targets.

There are methods to measure the phosphorylation state of various substrates to elucidate the phosphorylation pathways/targets. Mass spectrometry detects phosphorylated residues with a high sensitivity, but additional analyses, such as subtraction and tracing analyses, are needed to reveal changes in phosphorylation levels over time during intracellular events^[3]^. Other methods, such as reversed-phase protein arrays^[4]^ and antibody array measurements^[5]^, facilitate an accurate detection of phosphorylation, but the pathway analysis using these antibody-based measurements is dependent on the availability of specific antibodies, although their coverage has been recently improved^[6]^. In other words, there is a risk of arbitrary selection of appropriate methods by investigators in the measurements of protein phosphorylation.

Thus, we consider phosphorylation as a molecular event from the perspective of measurement data analysis, such as in data science and artificial intelligence (AI) technologies. Currently, the measurement of mRNA levels by microarrays and next-generation sequencers provides some important information on gene expression, while the measurement of DNA methylation levels provides important information on the regulation of gene expression. What is important about these measurements is that they are platform-type measurements, which allow measurements under various conditions by experimental investigators, and a large amount of information has been accumulated^[7]^. In addition, although not as standardized as the above-mentioned two types of measurements, it is possible to measure the amount of proteins and metabolites using mass spectrometers with a high precision, and some information on protein-protein interactions has also been accumulated^[8]^. Given this situation, it is expected that a platform-type measurement modality should be developed to investigate the phosphorylation state of the proteins responsible for signal transduction.

As a first step in the development of platform-based measurement modalities, we have developed a protein array that systematically and comprehensively measures the phosphorylation state of the proteins as well as a phosphorylation analysis system that also includes a mathematical system to analyze the measurement results. By using this platform, we have made novel discoveries regarding the efficacy of anticancer drugs^[9–10]^ and the visualized time-series changes in the epidermal growth factor receptor (EGFR)-mediated signal transduction^[11]^, and we also have compiled diverse phosphorylation variation patterns of kinase inhibitors^[12]^. In the current review, we introduce the workflow of our phosphorylation analysis system and illustrate an example of the target kinase estimation of inhibitors.

## The "Phospho-Totum": phosphorylation array analysis platform

The phosphorylation array analysis platform reproduces the phosphorylation state in a sample under certain experimental conditions using a proprietary protein array, in which the characteristics of the phosphorylation state are extracted through mathematical analyses.

The phosphorylation protein array that we have developed carries 1471 proteins on a glass plate, comprising 273 signaling pathways^[11]^ (***Supplementary Data 1***, available online). The pathways were selected by referencing the known signal transduction pathways of the Kyoto Encyclopedia of Genes and Genomes (KEGG)^[13]^ and Reactome^[14]^. The correlation between kinases and substrates in these pathways was investigated with reference to PhosphoSitePlus^[15]^, where 106 tyrosine kinases and 430 substrate proteins phosphorylated by these kinases (***Supplementary Data 2***, available online) had been found in 1471 proteins on the array. In addition, phosphorylation patterns had been measured and collected, when 167 commercial tyrosine kinase inhibitors (***Supplementary Data 3***, available online), including drugs, were administered to the phosphorylation arrays.

**Figure 1:**
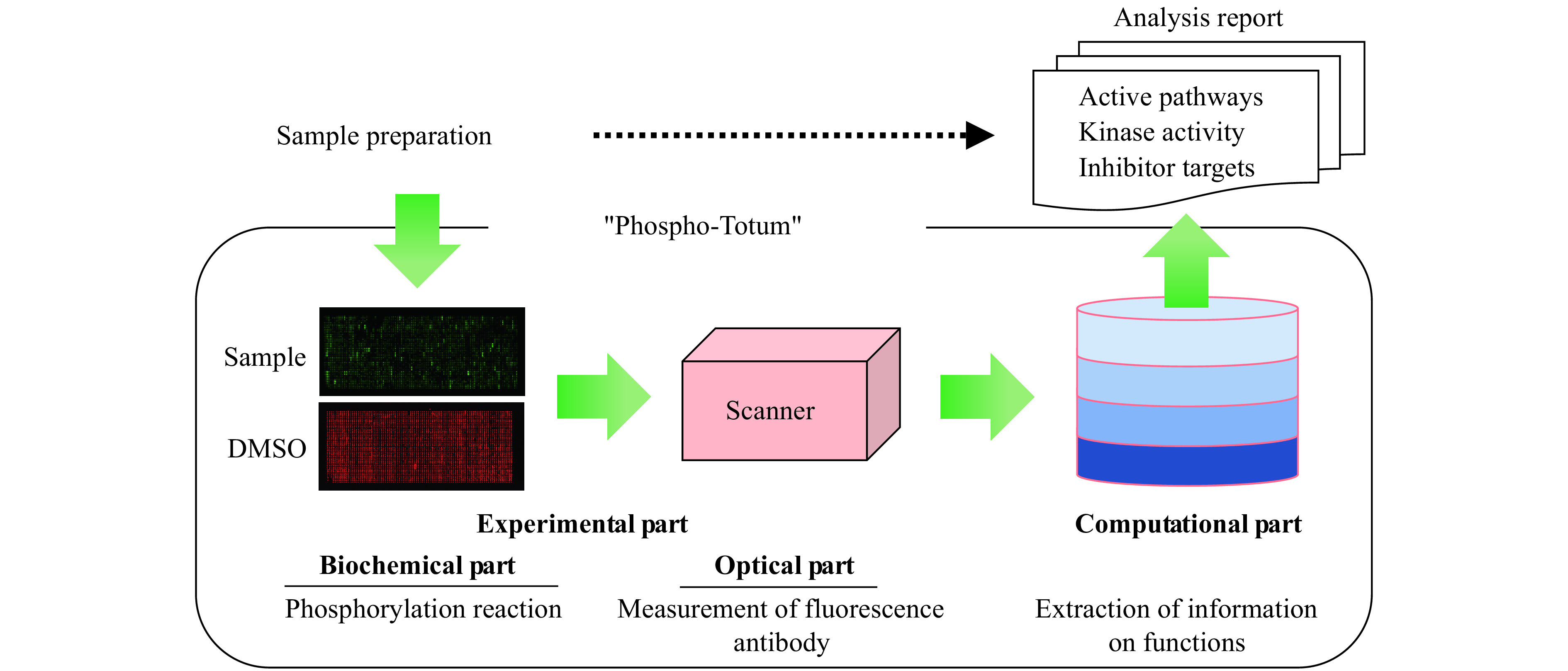
Overview of Phospho-Totum.

The workflow of the phosphorylation array analysis platform consists of the experimental measurement part of the phosphorylation reaction and its optical measurement, as well as the mathematical analysis part of the measured phosphorylation degrees (***Fig. 1***). When a sample is applied to the array, the proteins (substrates) on the array are phosphorylated according to the activation level of tyrosine kinases in the sample^[11–12]^. The levels of phosphorylation of these substrates are measured by the fluorescence intensity of the antibodies. In other words, the phosphorylation state of a protein in the cell is reproduced on the array according to the activation level of the kinase. The specific array preparation/measurement procedure is briefly summarized in ***Supplementary Data 4*** (available online). From the measured phosphorylation levels of the substrates, the system outputs the differentially phosphorylated substrates among the samples, the activated phosphorylation pathways, the kinase activation levels, and the similarity or non-similarity to known kinase inhibitors. Especially in the case of candidate tyrosine kinase inhibitor samples, the target kinase of the candidate drug is estimated from the above-mentioned analyses.

## AI-assisted analysis platform for analyzing the level of phosphorylation measured by the array

**Figure 2:**
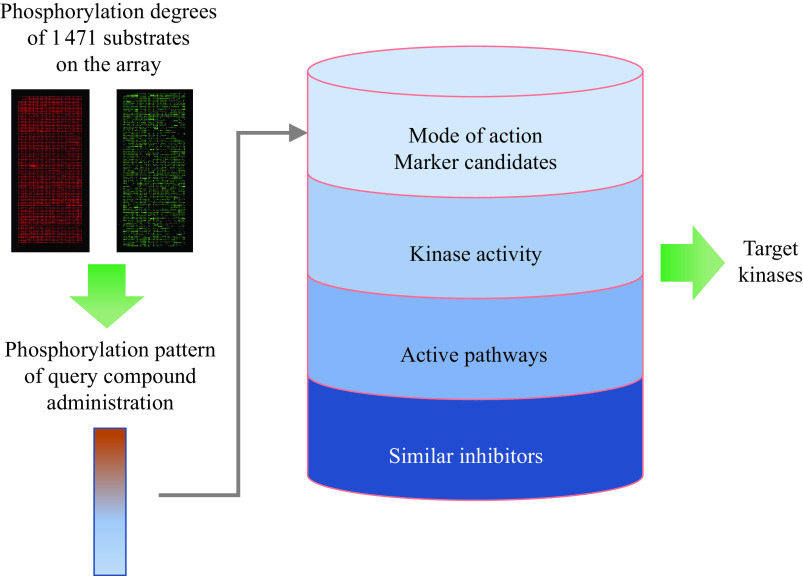
Data analysis workflow.

The experimental measurement section of the analysis platform reproduces the phosphorylation state of the proteins in the sample. In other words, the levels of phosphorylation of a large number of substrates measured under the same conditions are outputted as numerical data. From the phosphorylation data of the substrates, relevant information on the phosphorylation state in the sample is extracted by mathematical analyses (***Fig. 2***). Each part of the mathematical analysis is described below.

### Detecting differentially phosphorylated substrates under different conditions

First, the system estimates which substrates characteristically show variations in phosphorylation levels under the two compared conditions, such as healthy subjects and patients, or before and after compound administration.

The system employs three definitions for the variations between two conditions: the ratio of the measurements, the relative difference between the measurements by normalization to account for outliers in the measurements^[16]^, and the difference in the ranks of the substrates in the total measurements^[17]^. Based on these three definitions, the significance probability of each measurement value is estimated, respectively. A composite probability is then computed from the three significance probabilities^[18]^ and used as the significance probability of the substrate. This method allows the estimation of variations to be defined from multiple perspectives and eliminates arbitrary bias, because of the choice of a perspective from which to estimate significance.

The detected substrate groups may be useful in various directions. For example, these groups of substrates could be regarded as representatives of the conditions under which they were measured. Based on molecular functions of the substrate groups, they may be useful in elucidating biological functions of the sample under which conditions they were measured. Furthermore, because the substrates represent sample functions, they may also be regarded as candidate molecules for markers to classify the sample. In the case of pre- and post-drug administration data, they may considered to be candidates for drug efficacy markers; and in the case of data from healthy subjects and patients with a certain disease, they may considered to be candidates for diagnostic markers.

### Estimation of tyrosine kinase activation level

The novel array allows us to estimate the activation level of tyrosine kinases in a sample. Indeed, the inherent advantage of protein arrays is that changes in many protein groups are measured under the same conditions. Taking this advantage, we have developed a method to estimate the activation levels of 106 tyrosine kinases in a sample by mathematical analysis, based on the measured phosphorylation levels of 430 proteins, which are considered substrates for the 106 tyrosine kinases, among the 1471 substrates on the array^[9–12]^. The method is based on the following principles.

In general, one kinase phosphorylates different substrates, and the information on the pairs of kinases and their substrates has been accumulated in the database with the AI (neural net) model. With the input layer set to 1471 substrate phosphorylation levels and the output layer set to 106 kinase activation levels, the machine learning algorithm is applied as an intermediate layer based on the kinase/substrate-related knowledge. However, although kinase/substrate interactions are measured by various modalities, the content of kinase/substrate-related information varies. For example, in some information, kinase/substrate interactions are described only as binary relationships, while in others, the interaction information is measured as the amount of each protein. Therefore, the application of AI models requires validation of the kinase/substrate-related information based on the measurement of a larger number of array data.

As an initial step of estimation instead of AI network models, we assumed that, as a first approximation, the phosphorylation degrees of substrates were expressed by a linear combination of kinase activity as follows:

1 $ \left[\begin{array}{c}{p}_{1}\\ {p}_{2}\\  \vdots \\ {p}_{s}\end{array}\right]={a}_{1}\left[\begin{array}{c}{\delta }_{11}\\ {\delta }_{12}\\  \vdots\\ {\delta }_{1s}\end{array}\right]+{a}_{2}\left[\begin{array}{c}{\delta }_{21}\\ {\delta }_{22}\\  \vdots\\ {\delta }_{2s}\end{array}\right]+\cdots +{a}_{k}\left[\begin{array}{c}{\delta }_{k1}\\ {\delta }_{k2}\\  \vdots\\ {\delta }_{ks}\end{array}\right], $

where *p*_*i*_ (*i* = 1, 2, ···, *s*) and *a*_*j*_ (*j* = 1, 2, ···, *k*) are the phosphorylation degrees of the *s*-th substrate and the phosphorylation activity of the *k*-th kinase, respectively. *δ*_*ks*_ is the relationship between the *k*-th kinase and its *s*-th substrate, and is set as follows:

2 \begin{equation*}\begin{split} 
 {\mathrm{\delta }}_{ks}=\left\{\begin{array}{l}\frac{1}{{n}_{k}},\;{\mathrm{if}}\;{\mathrm{protein}}\;s\;{\mathrm{is}}\;{\mathrm{a}}\;{\mathrm{substrate}}\;{\mathrm{of}}\;k{\text{-}}{{th}}\;{\mathrm{kinase}},\\ \;\;\;\;\;\;\;{\mathrm{and}}\;{n}_{s}\;{\mathrm{is}}\;{\mathrm{a}}\;{\mathrm{total}}\;{\mathrm{number}}\;{\mathrm{of}}\;{\mathrm{substrates}}\\ \;\;\;\;\;\;\; {\mathrm{of}}\;k{\text{-}}th\;{\mathrm{kinase}},\\ \\ \\ 0,\; \;\;{\mathrm{otherwise}}.\end{array}\right. 
\end{split}\end{equation*}

In the present example, a total of 106 known tyrosine-kinases that possibly existed in cell lysate, and their substrates on the array, 430, as referred by PhosphositePlus^[15]^, were equipped on the array. Because the number of equations was not equal to that of variables in the linear system of equations, rigorous solutions for {*a*_*j*_} were generally not obtained. Therefore, we obtained approximate values of {*a*_*j*_} from Equation (Eqn.) (1) in two ways. One way is that Eqn. (1) is described as a matrix form as follows:

3 $ \vec{p}=\tilde{R} \vec{a} .$

In Eqn. (3), the problem was attributed to solving a system of the Moore-Penrose inverse matrix^[19]^, for the phosphorylation activity of kinases {*a*_*j*_} from the measured phosphorylation degrees of substrates {*p*_*i*_} and the information on kinase-substrate pairs {*δ*_*ks*_}.

Another way is to use a linear regression on Eqn. (1). The values of {*p*_*i*_} were measured, and those of {*δ*_*ks*_} were set based on knowledge of the kinase/substrate relationship. Then, the problem of finding the values of {*a*_*j*_} could be attributed to the problem of finding a solution to a linear system of equations in excess conditions. It could obtain an approximate solution by linear regression analysis.

### Estimation of pathway activation

The platform is equipped with a proprietary analysis tool to estimate activation/inactivation pathways. This method estimates the activation of each pathway from the phosphorylation levels of its constituent proteins measured under certain conditions, based on the consistency between the graph structure and the measured data^[20]^. Here, we briefly summarize the pathway screening as follows.

First, we constructed sets of pathway connectivity (binary data) with reference to the pathway databases KEGG^[13]^ and Reactome^[14]^. To estimate the activity by pathway screening for a directed acyclic graph (DAG) structure, we manually modified the original pathways according to the following rules.

1) The directions of arrows were set from the proteins in the plasma membrane to those in the nuclear membrane.

2) In the phosphorylation of a protein by a complex of proteins, the arrows were assumed from each of the constituent proteins in the complex protein to the protein.

3) In the pathway including a feedback loop, we separated one pathway into two pathways that were in the forward and backward directions.

Finally, we constructed 273 pathways of 1471 proteins with DAG structures.

Then, we calculated the graph consistency probability (GCP)^[20]^, which expressed the consistency of a given network structure with the monitored data of the constituent proteins in the present example. The consistency of a DAG structure, *G* (*V*_*i*_, *E*_*j*_), where *V*_*i*_ is a vertex (*i* = 1, 2, ···, *n*_v_) and *E*_*j*_ is an edge (*j* = 1, 2, ···, *n*_e_) in the graph, and the joint density function *f* (*X*_*i*_), corresponding to *V*_*i*_ for graph *G* with the measured data, is quantitatively expressed by the logarithm of the likelihood based on the Gaussian graphical model (GN: Gaussian Network)^[21]^, *i.e.*,

4 \begin{equation*}\begin{split} 
l\left({G}_{0}\right)=& \mathit{ln}\prod _{i=1}^{{n}_{v}}f\left({X}_{i}|pa\left\{{X}_{i}\right\}\right)\\
& =-\frac{1}{2}\sum _{i=1}^{{n}_{v}}\sum _{j=1}^{{n}_{i}}\Biggr\{\frac{1}{{\sigma }_{i}^{2}}\sum _{k=1}^{m}{\Biggr({x}_{ik}-\sum _{j=1}^{{n}_{i}}{\beta }_{ij}{x}_{kj}\Biggr)}^{2}+ \Biggr.\\
&\Biggr. \mathit{ln}\left(2\pi {\sigma }_{i}^{2}\right)\Biggr\}, 
\end{split}\end{equation*}

where *pa*{*X*_*i*_} is the set of variables corresponding to the parents of *V*_*i*_ in the graph, *x*_*ik*_ is the measured value of *X*_*i*_ at the *k*-th point, and *n*_*i*_ is the number of variables corresponding to the parents of *V*_*i*_. Here, the joint density function, *f* (*X*_*i*_), in the equation is expressed by the regression for the measured data. Because the likelihood depends on the graph size, we designed a simple procedure to transform the likelihood into the probability for the activation of the graph consistency with the data^[20]^. Indeed, we generated *N*_*r*_ networks under the condition that the networks shared the same numbers of nodes and edges as those of the given networks. Then, we defined GCP as follows:

5 $ GCP = \frac{{{N_s}}}{{{N_r}}}, $

where *N*_*s*_ is the number of networks with a log-likelihood larger than the log-likelihood of the tested network. In this study, *N*_*r*_ was set to 1000, and the *GCP* significance of the given network was set at 0.1.

## Variation patterns of known tyrosine kinase inhibitors

**Figure 3:**
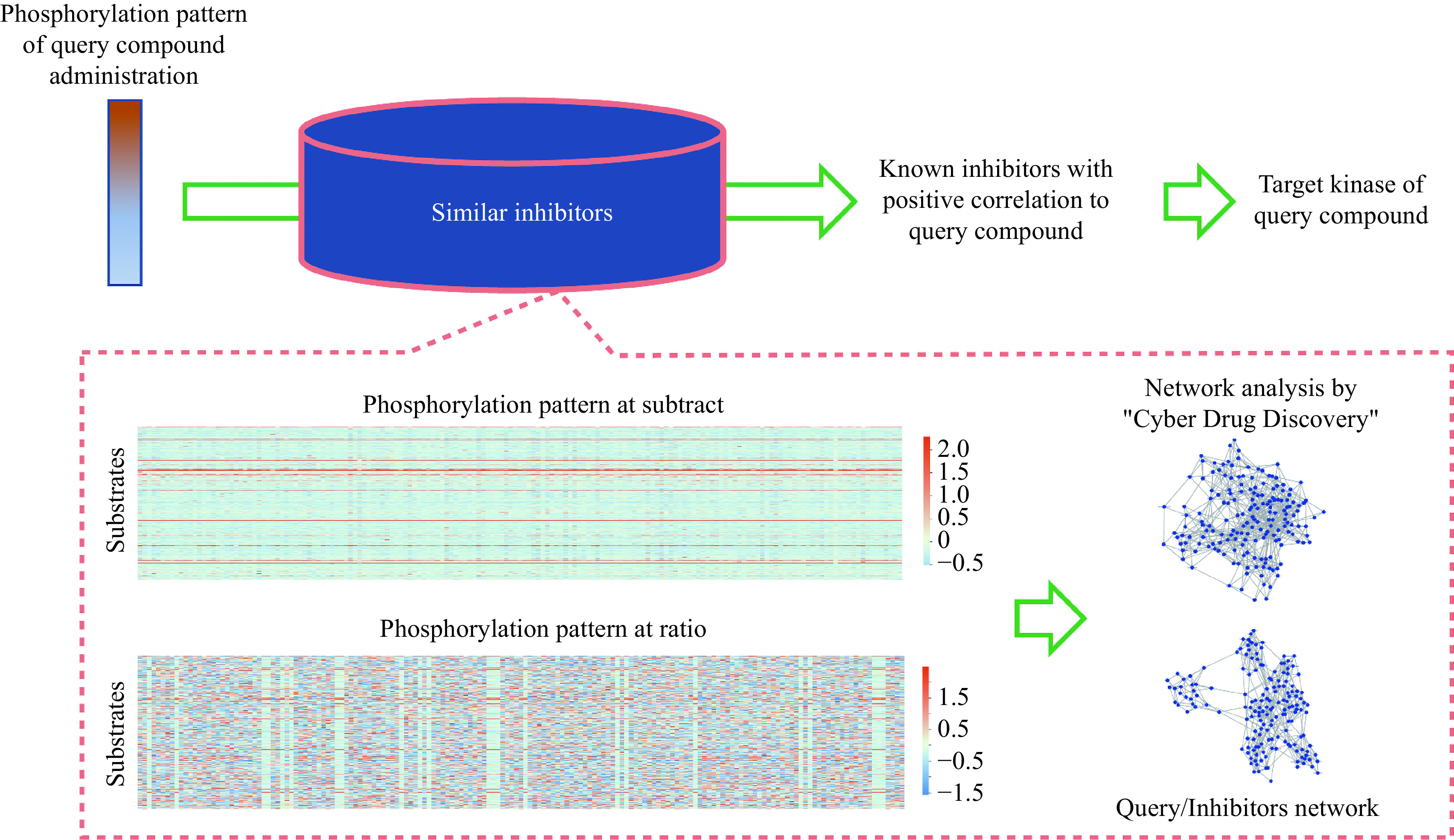
Identification of known inhibitors similar to query compounds by a network approach.

A variety of tyrosine kinase inhibitors are currently available for purchase, including compounds approved for pharmaceutical use. We prepared 167 tyrosine kinase inhibitors and measured their phosphorylation patterns in arrays (***Fig. 3***). In this process, the variation in phosphorylation levels was calculated in two ways: the ratio and difference before and after administration. The datasets of variation in the two ways were then compiled.

Based on this dataset, it is possible to estimate which tyrosine kinase inhibitors have similar phosphorylation patterns using a unique network technique. Furthermore, this estimation provides some useful information about compound targets. The network analysis based on phosphorylation patterns is as follows.

The correlation between gene expression and drug efficacy was uncovered by the Broad Institute in the connectivity map^[22]^ and the following Library of Integrated Network-Based Cellular Signatures (LINCS, https://lincsproject.org/) program. The direction of gene expression correlation between normal and disease was reversed in response to drug treatment, even for a few types of commercial cell lines, by the Gene Set Enrichment Analysis (GSEA)^[23]^, which estimates the distribution bias of a set of genes against the whole gene distribution. Because of the GSEA methodology, the Broad system needs approximately 1000 genes to estimate its reverse correlation. In contrast to the Broad system, we have developed another method for detecting the reverse correlation of gene expression between disease and drug efficacy, named "Cyber Drug Discovery"^[23]^. In our system, for example, we estimated that the drug candidates were negatively correlated with the disease by network analysis, followed by the detection of differentially expressed genes between normal controls and patients with a target disease by our original method^[16]^.

It is easy to apply our network analysis system for phosphorylation analysis. We may measure the phosphorylation variation pattern in a query compound and incorporate it into the known tyrosine kinase inhibitor variation pattern data as a query pattern. In other words, the newly measured data are considered the 168th dataset. A network analysis is performed on this dataset to estimate the association between the new sample and the 167 known tyrosine kinase inhibitors. If the new sample is found to be associated with a known tyrosine kinase inhibitor, then the properties of the new query compound are considered likely to be similar to the known tyrosine kinase inhibitor.

## Kinase inhibitor estimation

One of the goals of using this array is to determine whether a compound targets a kinase with an unknown function. To achieve this, the system has methods to estimate the target kinase from different perspectives.

Once the differentially phosphorylated substrates are identified, knowledge of the kinase/substrate interaction is used to determine which kinase substrate groups are specifically inhibited. Once the kinase activity is estimated, the group of kinases with a reduced activation is directly a candidate for the target kinase of the inhibitor. Once the active pathway is estimated, the group of substrates contained in the inactivated pathway is known, and the question of which target kinase may be answered from knowledge of the kinase/substrate interactions. Finally, if a similarity to a known inhibitor is found, the inhibitory target kinase of a similar known inhibitor is considered the inhibitory target kinase of the sample. As described above, this system provides multiple perspectives for selecting drug candidates with potential inhibitory target kinases.

## Visualization of the phosphorylation state

Apart from the target identification of query compounds, the system also provides tools for a clear understanding of dosing effects. Tools to visualize the entire pathway are also provided.

For the phosphorylation levels of 1471 proteins, which are the primary information obtained from the experimental measurements, a heatmap was created to visualize the overall changes. For the activation levels of the 273 pathways, it is necessary to use dedicated visualization software to obtain an overall picture. For this purpose, we further developed software that could seamlessly visualize the estimation results according to the framework of the cell structure, by using the localization information of the pathway component proteins and the binary correlation data of the pathway structure based on the activation pathway estimation results in the previous section. The 273 pathways were classified into 27 categories with reference to the Reactome Pathway Database^[14]^, and in each category, the activated pathways were visualized. Specific visualizations are illustrated in the next section.

## Example: data analysis of phosphorylation changes with dasatinib administration

An example of adapting the platform to an actual inhibitor target kinase is to measure the phosphorylation states before and after dasatinib administration. Dasatinib binds to the ATP-binding site of the BCR-ABL fusion protein and competes with ATP at the ATP-binding site. Dasatinib also binds SRC family kinases (SRC, LCK, YES, and FYN), c-KIT (KIT), ephrin (EPH) receptor A2 (EPHA2), and platelet-derived growth factor (PDGF)-β receptor (PDGFRB) to compete with ATP at the ATP binding site in the kinase domain of the above tyrosine kinase^[24]^. The approved indications of dasatinib are chronic myelogenous leukemia and Philadelphia chromosome-positive acute lymphoblastic leukemia. Using our analytical methods, we may examine whether our pre- and post-dose data predict the administered inhibitor dasatinib and whether the above target proteins may be identified as targets.

**Figure 4:**
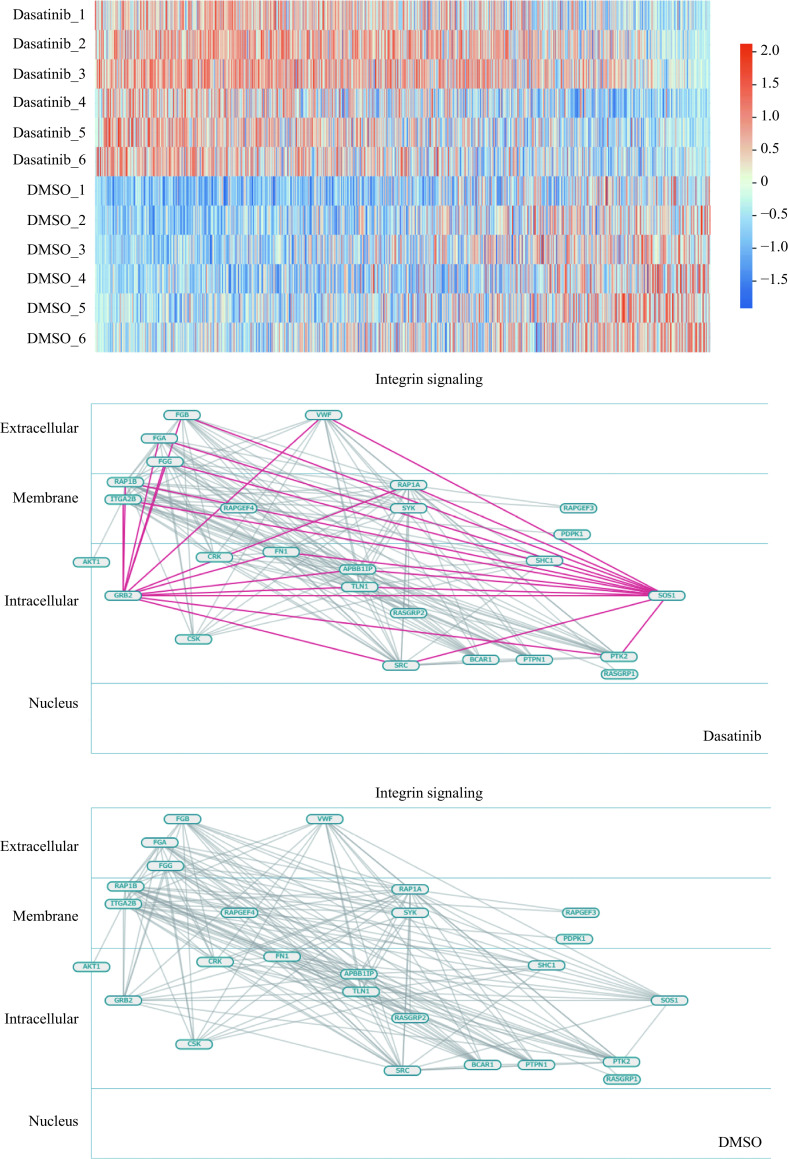
Visualization of active pathways by Phospho-Totum.

First, the visualization tool was used to intuitively capture the phosphorylation state changes before and after dasatinib administration. A heatmap of the changes before and after dasatinib administration is shown (***Fig. 4***). As is evident from the figure, different phosphorylation levels of some substrates on the array were detected before and after dasatinib administration. It was also observed that the inhibitor treatment with dasatinib did not increase the levels of phosphorylation of many substrates but rather decreased them. We visualized the activation of pathways categorized as "Integrin signaling" (***Fig. 4***), one of the 27 categories comprising 273 pathways. These integrin-related pathways were visible in each condition before and after administration. However, the integrin active pathway was only observed after treatment, not before treatment, indicating that dasatinib activated the integrin-related pathway. The other 26 categories were also visualized in the same way, so that the activation pathways before and after treatment may be intuitively understood.

**Figure 5:**
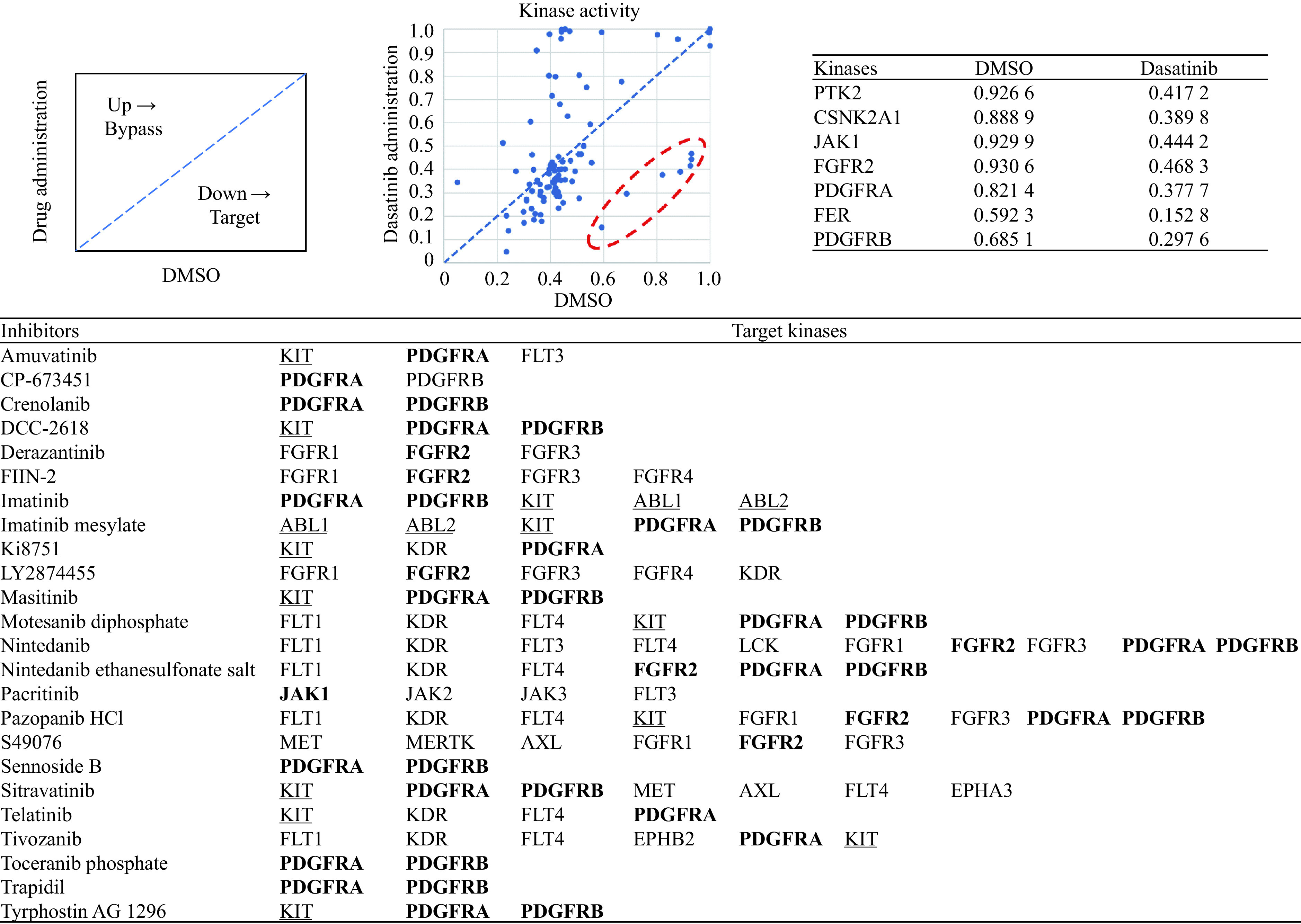
Illustration of target identification by kinase activity estimation in Phospho-Totum.

Based on these data, we could estimate the kinases that were inhibited. First, we selected kinases whose activity was decreased by dasatinib treatment (***Fig. 5***). We also estimated the activation level of the kinase in each of the DMSO and dasatinib conditions. When the activation level was lower in DMSO than in dasatinib, the kinase might be a target whose activity was suppressed by dasatinib, and vice versa, the kinase might be involved in bypass by dasatinib administration. As a result, we found that the activity was specifically decreased in seven kinases, among which PDGFRB, a known target of dasatinib, was found. Furthermore, with reference to the inhibitor/target-kinase correlation, inhibitors targeting these seven kinases were 24 out of 106. Many inhibitors showed kinases similar to those inhibited by dasatinib, such as ABL, KIT, and PDGFRB^[24]^.

**Figure 6:**
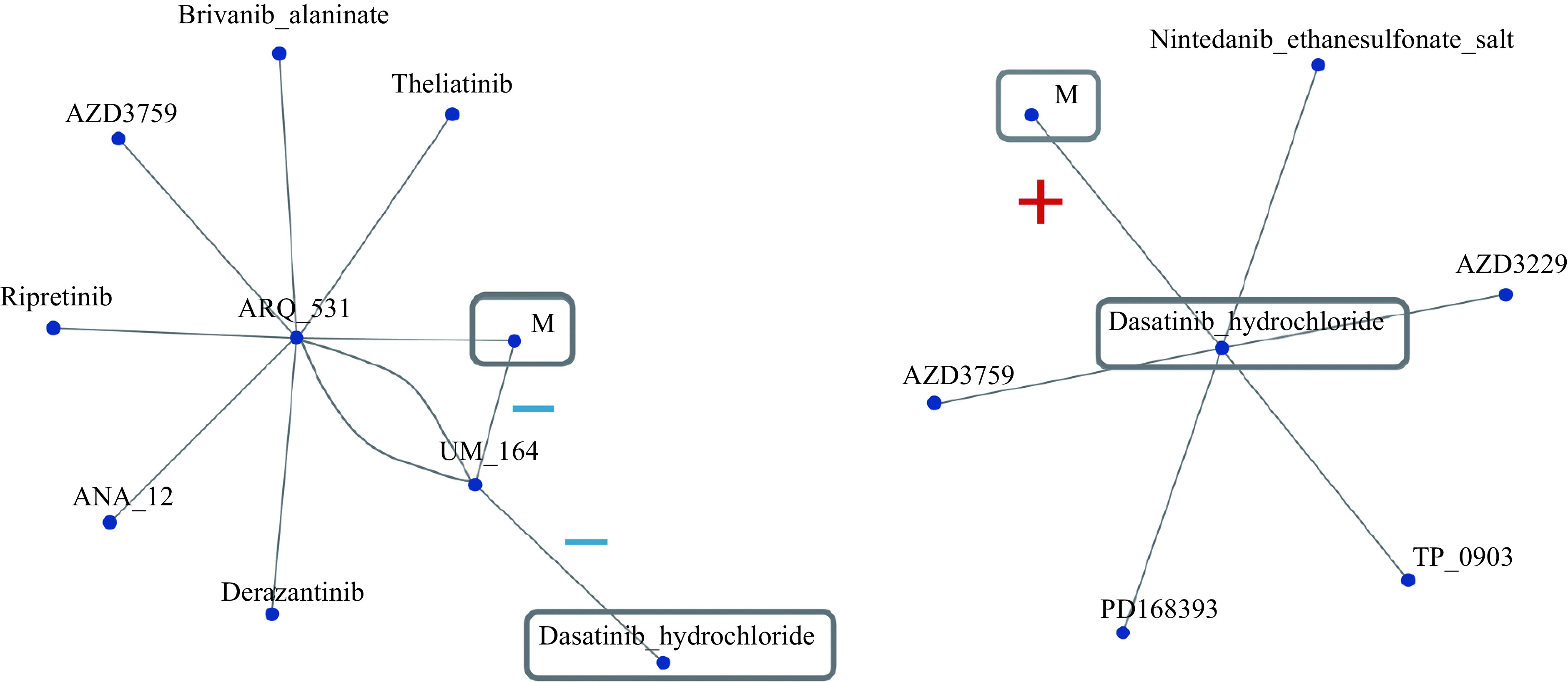
Illustration of target identification by network analysis in Phospho-Totum.

Inhibitors showing similar phosphorylation patterns were also estimated from the data before and after dasatinib treatment. Inhibitors showing correlated patterns were searched for by network analysis^[25]^ for the datasets of the variability ratios and differences before and after inhibitor treatment (***Fig. 6***). We extracted the network where the measured dasatinib ("M") was connected to the dasatinib ("Dasatinib_hydrochloride") stored in the datasets. As shown in ***Fig. 6***, the newly measured dasatinib was connected to dasatinib in the phosphorylation datasets. Indeed, in the raw subtraction data of phosphorylation, the newly measured dasatinib was connected to UM_164 with a negative correlation, and UM_164 was connected to dasatinib in the dataset with a negative correlation. In the ratio data of phosphorylation, the newly measured dasatinib was directly connected to dasatinib in the dataset set with a positive correlation. This indicated that dasatinib was successfully detected by this system and had the potential to identify the target of query inhibitors.

## Discussion

In the present review, we provide an overview of the phosphorylation analysis platform "Phospho-Totum" that we have developed. Using this platform, we may estimate the activation or inactivation pathways by compound administration and the activity of the kinase in any type of samples, such as blood and tissues. Additionally, a dataset of known tyrosine kinase inhibitors was used to estimate known compounds that show similar kinase activation levels to the administered compound. These results also indicate that our phosphorylation analysis platform provides useful information for target identification of compounds. For example, the performance of our system was evaluated by estimating the pathway activity of epidermal growth factor (EGF) stimulation and (EGFR) pharmacological inhibition^[11]^. As a result, by accurately measuring the phosphorylation levels of the constituent proteins on the array, the pathway activity upon EGF stimulation and EGFR inhibition was successfully traced along the time axis of the relevant pathway from the outer membrane to the nucleus.

In addition to drug discovery, this platform may also be useful in two additional tasks: the elucidation of disease mechanisms involving signal transduction and the discovery of disease and drug markers. Estimating the activation pathways and kinase activation levels in healthy subjects and patients helps to identify key molecular events involved in the pathophysiological process of disease. For example, using this analysis platform and patient-derived lung cancer cells, we found that activation of the insulin-like growth factor receptor type 1 pathway mediated by insulin-like growth factor 2 autocrine was a common clinically associated mechanism of the acquired resistance to osimertinib^[10]^. Furthermore, substrates that specifically fluctuate before and after the administration of drug candidates may be considered candidate markers. Exploring even more phosphorylation drug markers may provide an alternative to gene mutation-based drug efficacy diagnostic tools for individual patients. In sum, this platform will provide new answers to various questions with a different approach than conventional phosphorylation analysis modalities, such as antibody arrays and mass spectrometry.

In general, the AI approach requires larger amounts of data than statistical approaches. At this stage, the training data are not yet sufficiently developed to make the full-scale use of AI tools^[26]^. There was only one type of array, with only approximately 1000 arrays measured thus far, and furthermore, only 167 data pointed on kinase inhibitor administration at one time point (6 h after administration) and one concentration (optimal concentration of each drug); however, despite such a small amount of data, we were able to estimate the target with a high accuracy in the presented example. This will further enable the use of AI tools, if the measured data of the array of phosphorylation should be accumulated in the future. The present system is expected to evolve into a prediction system that may estimate targets based on the similarity of kinase activation and inhibition patterns, as well as disease or drug effect markers with a high accuracy, simply by inputting array measurement data.

## SUPPLEMENTARY DATA

Supplementary data to this article can be found online.
