# Supplementary material for: Phosphorylated protein chip combined with artificial intelligence tools for precise drug screening
Source: J Biomed Res. 2024 May 27;38(3):195–205. doi: 10.7555/JBR.37.20230082 (PMC11144935; doi:10.7555/JBR.37.20230082)
Supplement: Supplementary file 1 — Supplementary data to this article can be found online. [file jbr-38-3-195-S1.pdf]

Available online at www.jbr-pub.org.cn

Open Access at PubMed Central

Journal of Biomedical Research, 2024 0(000): 1–2

Supplementary Data

JBR

Phosphorylated protein chip combined with artificial intelligence tools for precise drug screening

Katsuhisa Horimoto[1,2,3,✉], Yuki Suyama[1], Tadamasa Sasaki[1], Kazuhiko Fukui[4], Lili Feng[5], Meiling Sun[6], Yamin Tang[5], Yixuan Zhang[7], Dongyin Chen[5,7,8,✉], Feng Han[5,7,8,✉]

[1]*SOCIUM Inc., Tokyo 1350064, Japan;*
[2]*International Medical Center, Saitama Medical University, Saitama 350-1298, Japan;*
[3]*Artificial Intelligence Research Center, National Institute of Advanced Industrial Science and Technology, Tokyo 1350064, Japan;*
[4]*Department of Informatics and Data Science, Sanyo-Onoda City University, Yamaguchi 7560884, Japan;*
[5]*International Joint Laboratory for Drug Target of Critical Illnesses, School of Pharmacy, Nanjing Medical University, Nanjing, Jiangsu 211166, China;*
[6]*Department of Physiology, School of Basic Medical Sciences, Nanjing Medical University, Nanjing, Jiangsu 211166, China;*
[7]*Gusu School, Nanjing Medical University, Suzhou, Jiangsu 215001, China;*
[8]*National Vaccine Innovation Platform, Nanjing Medical University, Nanjing, Jiangsu 211166, China.*

Supplementary Data 1: Names of 273 signaling pathways and their component proteins on the phosphorylation array (available online).

Supplementary Data 2: List of 106 tyrosine kinases and their target substrates on the phosphorylation array (available online).

Supplementary Data 3: List of tyrosine kinases measured by the phosphorylation array before and after the treatment (available online).

Supplementary Data 5: Visualization of activation pathways classified into 27 categories (available online).

Supplementary Data 6: The binary relationship between the component proteins in each pathway with the category (available online).

Supplementary Data 7: The estimation of pathway activations in each category, where the threshold of activation probability is set to be 0.2 (available online).

Supplementary Data 4: Construction of protein array for signal transduction

We adopted proteins synthesized using a wheat germ cell-free system as the substrate, which was previously shown to be suitable for phosphorylation assays[1]. Glutathione S-transferase (GST)-tagged proteins were synthesized as previously reported[2]. In brief, gateway entry clones from the Human Gene and Protein Database cDNA library[3] were inserted in the pEuGW-5FG expression vector with LR Clonase (Cat. #11791020, Thermo Fisher Scientific, Waltham, MA, USA). The PCR product from the LR reaction solution was used as a template for mRNA synthesis by RNA synthetase (Promega, Madison, WI, USA). Full-length GST-tagged proteins were then synthesized with the WEPRO7280G (Cat. #CFS-C7, CellFree Sciences, Ehime, Japan) wheat germ expression system. The synthesized proteins were spotted on glass slides by a Genex Arrayer (Cat. # Genex 2005 Arrayer, Kaken Geneqs, Chiba, Japan).

✉Corresponding authors: Katsuhisa Horimoto, Department of Research and Development, SOCIUM Inc., Aomi 2-4-7, Koto-ku, Tokyo 1350064, Japan. E-mail: katsuhisa.horimoto@socium.co.jp; Dongyin Chen and Feng Han, International Joint Laboratory for Drug Target of Critical Illnesses, School of Pharmacy, Nanjing Medical University, 101 Longmian Avenue, Jiangning District, Nanjing, Jiangsu 211166, China. E-mails: chendongyin@njmu.edu.cn (Chen) and fenghan169@njmu.edu.cn (Han).

Received: 07 April 2023; Revised: 07 September 2023; Accepted: 18 September 2023; Published online: 27 May 2024

CLC number: R91, Document code: A

The authors reported no conflict of interests.

This is an open access article under the Creative Commons Attribution (CC BY 4.0) license, which permits others to distribute, remix, adapt and build upon this work, for commercial use, provided the original work is properly cited.

© 2024 by Journal of Biomedical Research.

https://doi.org/10.7555/JBR.37.20230082

Approximately 500 nL synthesized protein crude solution was spotted at a density of 4 704 spots per slide. Every protein was spotted six times (n = 3) on the same slide. Before spotting, the glass slide was coated with 0.1 mmol/L sSMPB (Thermo Fisher Scientific) in phosphate buffer saline and 50 mmol/L reduced glutathione (GSH) solution in 50 mmol/L Tris-HCl, following the manufacturer's instructions. During spotting, the humidity was maintained at 40%–60%, and each spot was kept moist, as confirmed by microscopic observation of liquid droplets. After spotting, array slides were stored at −80 ℃ in blocking buffer (50 mmol/L Tris-HCl [pH 7.3], 200 mmol/L NaCl, 0.08% [v/v] Triton X-100, 5% PhosphoBlocker, 5 mmol/L GSH [pH 7.3], 25% [v/v] glycerol, and 2 mmol/L DL-dithiothreitol [DTT]).

Preparation of cell lysates

A431 cells (Cat. #EC85090402-F0, KAC Co., Ltd., Kyoto, Japan) were seeded at 3 × 10^6 cells per mL in 10% fetal bovine serum, 1× non-essential amino acids, and Eagle's minimum essential medium at 37 ℃ with 5% CO_2. Cells were treated with 10 µmol/L Dasatinib (Selleck Chem, Houston, TX, USA) or DMSO for 2 h. After treatment, we adopted M-PER for whole-cell lysate preparation for the following phosphorylation measurements. Cell lysates were centrifuged at 14 000 g at 4 ℃ for 10 min. Supernatants were then stored at −80 ℃ until the measurement.

Phosphorylation activity measurement

For the cell lysate assay, the amount of total protein in the cell lysate was quantified by a bicinchoninic acid assay (Thermo Fisher Scientific). For sufficient phosphorylation detection, all analyses were performed by loading cell lysate containing 10 µg total protein on an array slide by the following steps.

1. The lysate was suspended in kinase reaction buffer (25 mmol/L Tris-HCl [pH7.5], 5 mmol/L β-glycerophosphate, 0.1 mmol/L Na_3VO_4, 10 mmol/L $MgCl_2$, 1 mmol/L ATP, and 2 mmol/L DTT) and applied to the array slide at 30 ℃ for 3 h.

2. Kinase reactions were terminated with termination buffer (50 mmol/L ethylene diamine tetraacetic acid, 10 mmol/L HEPES-NaOH [pH7.4], 150 mmol/L NaCl, and 0.05% [v/v] Tween-20) at 30 ℃ for 5 min.

3. After washing twice with Tris-buffered saline T (TBST) for 5 min, phosphorylated tyrosine residues were detected by 4G10 (Cat. #32160702, Merck, Darmstadt, Hessen, Germany) for 1 h and diluted 1 : 2 000 in 5% PhosphoBlocker (Cell Biolabs, San Diego, CA, USA)/TBST.

4. After washing with TBST three times for 5 min each, secondary staining was performed with mouse IgG antibody and Alexa Fluor 647 (Thermo Fisher Scientific) diluted 1 : 2 000 in 5% PhosphoBlocker/TBST for 1 h. Array slides were then washed with TBST three times for 5 min each and then with distilled water for 5 min.

5. The slides were then dried by centrifugation (800 g, 3 min).

References

[1] Takeda H, Kawamura Y, Miura A, et al. Comparative analysis of human SRC-family kinase substrate specificity *in vitro*[J]. *J Proteome Res*, 2010, 9(11): 5982–5993.

[2] Maruyama Y, Wakamatsu A, Kawamura Y, et al. Human gene and protein database (HGPD): A novel database presenting a large quantity of experiment-based results in human proteomics[J]. *Nucleic Acids Res*, 2009, 37(suppl_1): D762–D766.

[3] Goshima N, Kawamura Y, Fukumoto A, et al. Human protein factory for converting the transcriptome into an *in vitro*-expressed proteome[J]. *Nat Methods*, 2008, 5(12): 1011–1017.
